# Physical and social environmental characteristics of physical activity for Mexican-origin children: examining differences between school year and summer perceptions

**DOI:** 10.1186/1471-2458-14-958

**Published:** 2014-09-16

**Authors:** M Renée Umstattd Meyer, Shana M Walsh, Joseph R Sharkey, Grant B Morgan, Courtney C Nalty

**Affiliations:** Department of Health, Human Performance & Recreation, Baylor University, One Bear Place #97313, Waco, TX 76798-7313 USA; Department of Health Promotion and Community Health Sciences, Program for Research and Outreach-Engagement on Nutrition and Health Disparities, School of Public Health Texas A&M Health Science Center, TAMU 1266, College Station, TX 77843 USA; Department of Educational Psychology, Baylor University, One Bear Place #97301, Waco, TX 76798-7301 USA; Baylor College of Medicine, McNair Building, 7200 Cambridge St., MCHP-1000 MS: BCM390, Houston, TX 77030 USA

**Keywords:** Health disparities, Minority health, Seasonality, Environment, Social support, Exercise

## Abstract

**Background:**

*Colonias* are substandard residential areas along the U.S.-Mexico border. Families of Mexican-origin living in *colonias* face health burdens characterized by environmental and socioeconomic hardships. Mexican Americans and low-income families, including *colonias* children, do not frequently participate in physical activity despite the known link to disease risk reduction. For *colonias* children, schools are the most commonly reported location for physical activity. School closures and extreme temperatures during summer months create a need to explore seasonal differences in environmental supports and barriers in this population. The purpose of this study was to examine the effect of seasonality on perceived environmental barriers, opportunities, and social support for physical activity among *colonias* children. As a secondary aim, mother-child discordance for each factor was analyzed.

**Methods:**

Promotora-researchers recruited mother-child dyads (n=101 dyads, n=202 participants) from *colonias* in Hidalgo County, Texas. Mothers and children were separately administered surveys at two time points to capture perceived barriers, opportunities, and social support for physical activity (school-year: February-May; summertime: July-August). Summative scores for each outcome were calculated and three multilevel longitudinal models for continuous outcomes were examined; children were nested within households. Mother-child discordance was measured using Cohen’s Kappa statistic.

**Results:**

Physical activity barriers and environmental opportunities (household and neighborhood) increased from school-year to summer by 1.16 and 2.83 points respectively (*p*≤0.01), after adjusting for covariates. Significant predictors of increased barriers included household income of >$900/month and having more household members. Children of mothers with significant others who were employed part-time or full-time saw significant decreases in barriers. Mother-child agreement of barriers, environmental opportunities, and social support across seasons was slight to fair (range: median κ=0.047 to κ=0.262).

**Conclusions:**

These results suggest a complex relationship between dimensions of economic hardship (employment status, household income, etc…) and perceived opportunities and barriers of children’s physical activity engagement during the school-year and summer. In this study, both barriers and opportunities increased from school-year to summer, further demonstrating that interactions among these characteristics need to be better understood and addressed when considering physical activity initiatives for *colonias* and other Mexican-American children, specifically during summer when school-based physical activity resources are unavailable.

## Background and purpose

Sedentary behavior is rapidly emerging as an important issue in public health, and concern over the number of children and adolescents adopting sedentary lifestyles has grown in recent years [1, 2]. This is largely due to the deleterious health outcomes associated with sedentary behaviors, and the particular significance of time spent being sedentary among children and adolescents [3, 4]. In addition to a greater risk of childhood obesity and health risks in adulthood, research indicates that the trend of decreased levels of physical activity and increased levels of sedentary behaviors is threatening the persistent increase in life expectancy enjoyed over the past century [5]. Children who do engage in regular physical activity benefit from improved bone health, cardiorespiratory and muscular fitness, decreased levels of adiposity and thus reduced future health risks, as well as psychological benefits including increased self-esteem levels and reduced symptoms of depression [6, 7]. Despite the known benefits of physical activity and the recommendation for all children to engage in moderate to vigorous physical activity for 60 or more minutes each day, many U.S. children do not meet the physical activity recommendation and are spending an increased amount of time in sedentary behaviors [8]. Physical activity has been attributed to varied personal, social, environmental, and economic factors [6, 9]. Given its significance, better understanding of the contributing factors to childhood physical activity participation has the potential to result in more effective physical activity initiatives.

The environmental influence on physical activity participation in youth has been well established. A review conducted by Davison and Lawson [9] explored the relationship between environment features and children’s physical activity and found that environmental supports for children include sidewalks in their neighborhood, destinations to walk to, fewer uncontrolled intersections, and low traffic density [9]. Availability of play equipment and permanent activity structures in school play areas were also associated with higher levels of physical activity [9]. Environmental barriers to physical activity participation in children included higher neighborhood crime rates and the perceived presence of roaming dogs [9].

Seasonality may also affect physical activity behaviors. Levels of physical activity have been established to vary with seasonality, with poor or extreme (e.g., very hot, very cold, etc…) weather identified as a barrier to physical activity participation [10–12]. In Canada, where extreme weather conditions exist in during the winter, Canadian adults were found to be 86% more likely to engage in leisure-time physical activity in the summer than in the winter [13]. In areas where extreme weather exists in the summer, reverse trends are likely where activity is more probable during the season with less extreme weather conditions. In addition, many physical activity interventions occur during the school year despite evidence showing greater weight gain during summer months for some populations of children [14, 15]. Given this disconnect, it is important to understand the role of seasonality with regards to physical activity participation of children. This information could help health promotion and physical activity practitioners better understand seasonality trends and subsequently plan more effective future physical activity initiatives.

Mexican-origin families living in *colonias* along the Texas-Mexico border are impacted by seasonality, specifically extreme summer weather conditions. During the summer months (June- August), average high temperatures in Hidalgo County, Texas range from 97-99 °F, and record high temperatures have reached 110 °F [16]. Previous research within *colonias* communities cited schools as the most frequent location for children’s physical activity, and school buses as the most common transportation option used by children to access physical activity opportunities [17]. However, schools are not in-session during summer months, and school buses are not available as a means of transportation. In addition, there are limited physical activity programs, especially programs with no charge, which could alter environmental support and/or barriers to physical activity [17].

**Figure 1:**
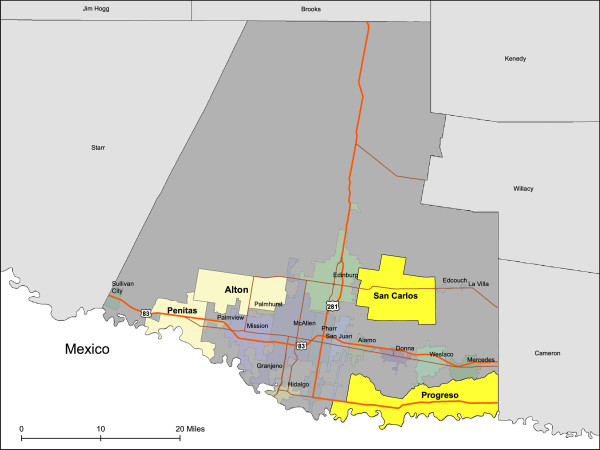
**Map of Hidalgo County, Texas colonias areas.**

*Colonias* are mixed-quality residential areas along U.S.-Mexico border and are characterized by inadequate basic services including electricity, water, sewage, fire protection, policing, schools, and health care [18, 19]. There are more than 2,500 *colonias* spanning the entire U.S.-Mexico border, but the majority exists within the state of Texas [18, 19]. In Hidalgo County alone, there are approximately 860 *colonias* providing homes to over 150,000 residents [19]. Please see the map below (Figure 1). *Colonias* residents are predominately of Mexican heritage and most encounter severe poverty and extremely high rates of unemployment [18]. *Colonias* residents also face great health disparities resulting in disproportionately high rates of obesity, type 2 diabetes, and related illnesses [20, 21]. Additionally, the Mexican-origin population is the fastest growing racial/ethnic group in the U.S. and much of this growth is occurring in *colonias* and in other new immigrant destinations throughout the country [22, 23]. Despite the link between physical activity and reduced disease risk being well established for people of all age groups and backgrounds, research shows that few Mexican-Americans and low-income families, including children*,* engage in regular physical activity [24, 25].

The purpose of this study was to examine the effect of seasonality on environmental factors of physical activity among Mexican-origin children residing in *colonias* in the Hidalgo County, Texas border region, specifically, perceived environmental barriers to physical activity, perceived opportunities for physical activity, and perceived social support for physical activity. As a secondary aim, child-mother agreement across the factors was examined. Given the nature of perception, perceived environmental supports and barriers may differ between mothers and children. Looking at mother-child agreement across factors can aid in understanding the complete picture. Consistent with previous findings, the authors hypothesize that extreme summer temperatures characteristic of *colonias* will serve as a barrier to physical activity, and be compounded with the elimination of school-based physical activity opportunities during the summer months [10–13, 17].

## Methods

### Setting

Four large geographic areas of *colonias* located in Hidalgo County along the Texas-Mexico border in the Lower Rio Grande Valley were included in the present analysis. Hidalgo County, Texas is 1,571 square miles in size and has a population of 774,769, of which 91% are Hispanic or Latino, 35% live below the poverty level, and the median household income is $33,218 (2008-2012) [26]. Selection methods of these four areas have been previously described [27–31]. *Colonias* residents are primarily of Hispanic origin, encounter numerous economic and locational disadvantages, characterize a difficult-to-reach population, and may mirror archetypes for emerging new-immigrant destinations elsewhere in the U.S. [18, 31].

### Study sample

*Promotora*-researchers (State of Texas certified indigenous community health workers trained in research methods) recruited 106 Mexican-origin mother-child dyads. Inclusion criteria required one child (age 6-11 years) who resided full-time from each participating home, and each child’s mother. Complete data were available on 101 dyads (202 participants).

*Promotora*-researchers explained aspects of the study (assessments, confidentiality, and financial incentive) to mothers, who provided consent for themselves and for her child while children provided assent to participate. All materials and protocols were approved by the by the Texas A&M University and Baylor University Institutional Review Boards.

### Data collection

All data were collected using *promotora*-administered Spanish-language surveys within participants’ residences across two waves between February and August 2011. Baseline (school-year) surveys were completed February – May 2011, while follow-up (summer time) visits occurred between July – August 2011. Following instrument translations by a team of bilingual (native Spanish) translators and verification by a team of *promotora*-researchers to ensure semantic, conceptual, and normative equivalence, data were collected in Spanish, the native language of participants. Mothers and children were interviewed separately to avoid bias.

### Measures

Each mother and child completed three physical activity surveys at two points in time (school-year and summer): 1) perceived environmental and personal barriers to physical activity, 2) perceived opportunities for physical activity in the home and neighborhood, and 3) perceived social support for physical activity. Socio-demographic information was also collected for each participant.

### Perceived barriers

Development of barrier items has been reported elsewhere [17]. In summary, 28 barrier items were developed to capture environmental, interpersonal, and intrapersonal level barriers using current physical activity literature, physical activity barrier scales, and feedback from *promotora*-researchers [32–34]. Mothers reported their perception of their child’s barriers and children reported what they believed to be barriers to engaging in physical activity. Both mothers and children were instructed to select all barriers within the checklist that were applicable to her/him by selecting a response option of present or not present (yes or no), for a possible summative score ranging from 0-28.

### Perceived opportunities

**Figure 2:**
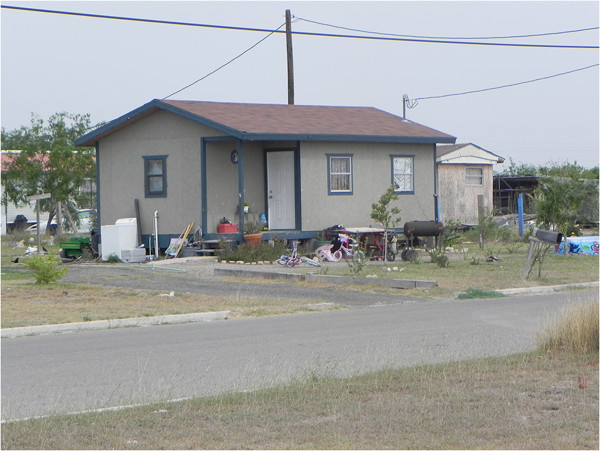
**Colonias residence with physical activity opportunities.**

Perceived opportunities for physical activity in the home and neighborhood were measured using 16-items for home opportunities and 21-items for neighborhood opportunities. Development of these items has also been previously reported [17]; however a brief description is included here. Items were developed using current physical activity environmental literature, established environmental assessment instruments, and visual scans of 32 *colonias* conducted by the researchers in 3 areas of Hidalgo County in South Texas [32, 35–37]. Mothers and children each reported their own perceptions of physical activity opportunities for their children or themselves, respectively. Similar to barriers, both mothers and children were instructed to select all opportunities within the checklist that were applicable to her/him by selecting a response option of present or not present (yes or no), for a possible summative score ranging from 0-37. See Figure 2 for an example of a *colonias* residence and physical activity opportunities.

### Social support

Children reported their perceptions of social support received from their parents and mothers reported perceptions of their provision of social support to their children. Children’s perceptions of parental social support for physical activity were measured with three items using a 7-point Likert scale of frequency to understand how often social support (in the form of parents being physically active with their children, encouraging their children to be physically active, and providing transportation for physical activity) was provided in the previous seven days (0-7 times) [38, 39]. Provision of social support for children’s physical activity was also examined using 3 items to measure the frequency of the mother’s demonstration of social support for her child. These items were adapted from Trost and colleagues’ (2003) original five-item scale [40]. Specifically, items ascertained whether the mother reported supplying transportation to and from physical activity/sport options for her child, exercising with her child, or orally reinforcing that physical activity is “beneficial” or “good” to her child. Although Trost’s original scale used a 5-point Likert scale ranging from “none” to “daily”, in this study each mother reported how many times she provided these types of social support to her child in the previous seven days (0-7). Possible summative scores for both mothers’ provision of social support and children’s receipt of social support ranged from 0-21.

### Analysis

Data were entered into Access databases and reviewed for accuracy by independent researchers. Following database merging and thorough data cleaning procedures, independent summative scores for each measure of interest were created for mothers and children for each time point. Outcomes of interest for each model were interaction of the summative score with the effect of seasonality (wave 1: school-year or 2: summer). Three multilevel longitudinal models for continuous outcomes, in which children were nested within households to account for both child and mother responses, were built to account for correlation inherent in repeated measurements and shared living environments using SAS (v. 9.3, 2012) MIXED procedure. Each model was adjusted for baseline demographic covariates (disclosed by mothers), including self-reported race/ethnicity (Hispanic, Mexican, Mexican-American), birth country (Mexico vs. U.S.), marital status (married/living with partner, not married vs. single/widowed/divorced or separated), mother’s education, total household income (<$500/month, $500-$899/month, ≥$900/month), employment status (mother; spouse or partner: full-time, part-time, not working), household size, and availability of personal transportation during the day. Employment status was determined by a single question asking “In your household who works for wages?” with the following categories available for response: you, spouse or partner, other adults, children. Spouse or partner employment was included for all mothers responding to this item regardless of marital status. Mother-child discordance for physical activity barriers, opportunities, and social support was measured using Cohen’s Kappa coefficient (κ) for each item. Cohen’s Kappa is a statistical measure of inter-rater agreement which takes into account agreement by chance (4). The level of precision, or amount of mother-child agreement, was assessed through criteria established by Landis and Koch, who ascribe κ of <0.00 as “poor”, 0.00-0.20 as “slight”, 0.21-0.40 as “fair”, 0.41-0.60 as “moderate”, 0.61-0.80 as “substantial”, and 0.81-1.00 as “almost perfect” (5). All analyses were performed using SAS (v. 9.3, 2012) [41].

## Results

**Figure 3:**
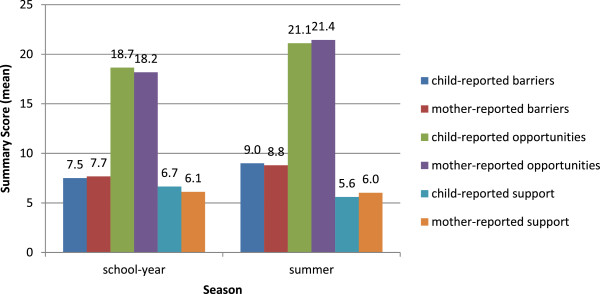
**Mean summary scores for physical activity opportunities, barriers, and social support reported by mothers and children during the school-year and summer.**

Among this sample of limited-resource, Mexican-origin families living in Texas border *colonias* (n = 101 dyads; n = 101 children, n = 101 mothers), 100% of the families were of Mexican heritage. Mothers were on average 35 years of age and 41.3% of mothers rated their health as fair (0% poor, 2% excellent). Eighty-one mothers were married or living with a partner but not married; although 99 mothers reported an employment status for their spouse or partner. Children were on average 9 years of age and 39.4% of children reported their health as very good or excellent, with only 17% reporting fair health and none reporting poor health. Please see Table 1 for additional baseline demographic information. Frequencies of the dependent variables of interest for school-year and summer as reported independently by mothers and children are displayed in Figure 3.

**Table 1:** **Baseline demographic characteristics (n = 101 dyads; n = 101 mothers, n = 101 children)**

Variables	
	*Mean* ± *standard deviation*
**Age (years)**	
Mothers *(n = 101)*	34.7 ± 6.9
Children *(n = 101)*	8.9 ±1.5
**Education completed (years)**	
Mothers *(n = 101)*	8.7 ± 3.4
Children (*n = 100)*	3.2 ± 1.4
**Household residency (n = 101)**	
Adults per household	2.2 ± 0.9
Children per household	3.5 ± 1.3
**BMI**	
Mothers *(n = 97)*	32.6 ± 7.1
	*Frequency* ^a^
**Sex of children (n = 101)**	
Female	58
Male	43
**Mother’s country of birth (n = 101)**	
Mexico	87
U.S.	14
**Mother’s marital status (n = 101)**	
Married	61
Living with partner	20
Divorced/Separated	9
Single	7
Widowed	4
**Mother’s race/ethnicity (n = 101)**	
Hispanic	29
Mexican	35
Mexican American	37
**Total household income (n = 95)**	
<$500/month	27
$500-$899/month	43
≥$900/month	25
**Employment**	
*Mother (n = 100)*	
Part-time	15
Full-time	10
Not employed	75
*Spouse or partner (n = 99)*	
Part-time	27
Full-time	43
Not employed	29

^a^Total number may not always equal total sample because of missing data.

### Barriers to physical activity

Over time, from school-year to summer months, perceptions of children’s barriers to physical activity increased by 1.16 points (*p* = 0.012), after holding constant demographic characteristics (including household income, work status of mothers and significant others, household size, mother’s race/ethnicity, mother’s country of birth, mother’s marital status, and mother’s education) as well as household car availability during the day. Factors that either increased or decreased barriers to children’s physical activity from school-year to summer included total household income, household size, and work status (full time, part time, or not employed) of mothers’ significant others. A total household income of greater than $900 per month as compared to < $500 was associated with a 1.23-point increase in children’s barriers to physical activity (*p* = 0.038) after adjusting for all covariates. Having more members in a household was associated with a 0.38-point increase in barriers to physical activity (*p* = 0.015) after adjusting for all covariates. Mothers’ significant others’ employment status was significantly associated with decreased barriers for children; where part-time employed significant others decreased children’s barriers by 1.58 points when compared to unemployed, and significant others employed full-time decreased children’s barriers by 1.68 points as compared to unemployed. Slight mother-child agreement was observed for children’s barriers to physical activity in the school-year (median κ = 0.12) and summer (median κ = 0.05). See Table 2 for kappa coefficients comparing mother-child agreement for all items.

**Table 2:** **Item discordance between mother and child reports (Cohen’s Kappa coefficients)**

Opportunities	*Spring*	*Summer*	Barriers	*Spring*	*Summer*
H-Trampoline	0.68	0.86	No transportation	0.18	0.07
H-Weight machine	0.27	0.42	Dogs	0.00	0.04
H-Balls	0.28	0.12	Energy	-0.03	0
H-Functional bball hoop	0.43	0.78	Traffic	0.17	0.06
H-Bikes	0.34	0.34	Crime	0.25	0.00
H-Swing set	0.62	0.88	Motivation	0.11	-0.06
H-Scooter	0.26	0.55	No place like a park	0.29	0.08
H-Volleyball	0.48	0.20	No partner	0.34	-0.04
H-Toy wagon	0.14	0.11	Immigration status	-0.02	0
H-Tire swing	0.23	0.54	Kidnappings	0.02	-0.08
H-Tires to roll	0.19	0.27	Time	0.16	0.06
H-Push car	0.19	0.29	Small kids at home	-0.02	-0.03
H-Play car	0.23	0.27	Adequate clothing	0.19	0.09
H-Patio	0.10	0.12	Can’t leave house	-0.02	-0.06
H-Paved driveway	0.52	0.59	No sidewalks	0.20	0.12
H-Pool	0.30	0.40	Trash	0.04	0.10
N-Trampoline	-0.08	0.17	No exercise place	0.26	0.19
N-Block	-0.08	0.01	No fenced space	0.03	0.04
N-Soccer field	0.04	0.26	No street lamps	0.12	0.05
N-Balls	-0.02	0.19	Farm animals	-0.04	0.30
N-Functional bball hoop	0.39	0.02	Gangs	-0.05	0.17
N-Kids in streets	-0.08	0.32	Fear of hurting	-0.05	0.25
N-Bikes	-0.02	-0.02	Not encouraged	0.14	-0.07
N-Good streets	0.11	0.31	Not fun	0.06	-0.11
N-Swing sets	0.01	0.36	Asthma	0.92	0.39
N-Walking route	-0.01	0.14	Heat	0.13	0.03
N-Volleyball	0.01	0.17	Bad weather	0.11	0.03
N-Parks	0.48	0.32	N-graffiti	0.48	0.41
N-Patios	-0.06	0.66			
N-Open spaces/fields	-0.00	0.19			
N-Paved roads	0.13	0.13			
N-Tires to roll	0.07	0.38	**Social support**	*Spring*	*Summer*
N-Kids playing	0.00	0.03	Parents exercise w/ you	0.14	0.14
N-Rec building	0.48	0.21	Transportation	0.08	0.11
N-Playground	-0.09	0.26	Encouragement	0.08	-0.06
N-Stoplights	0.11	-0.03			
N-Pool	0.17	0.21			

*H* = Home, *N* = Neighborhood, *bball* = Basketball, *w/* = With.

### Opportunities for physical activity

From school-year to summer, children’s opportunities for physical activity increased by 2.83 points (*p* < 0.001), after controlling for demographic covariates. Yet, in the longitudinal model the only variable that approached significance was mother’s country of birth, where being U.S. born was associated with a 2.10-point increase in opportunities from school-year to summer (*p* = 0.06) after adjusting for covariates. Slight mother-child agreement was observed for reports of physical activity opportunities in the school-year (median κ = 0.14) and fair agreement was observed in summer (median κ = 0.26). When examining mother-child agreement for home physical activity opportunities and neighborhood physical activity opportunities separately, agreement was much more likely for home opportunities. Fair agreement was observed for home physical activity opportunities in both the school-year (median κ = 0.28) and summer (median κ = 0.37), where only slight agreement was observed for neighborhood physical activity opportunities in the school-year (median κ = 0.01) and summer (median κ = 0.19). See Table 2 for kappa coefficients comparing mother-child agreement for all items.

### Social support for physical activity

Although not statistically significant, children’s social support to engage in physical activity provided by parents decreased from school-year to summer months by 0.54 points (*p* = 0.209), after controlling for demographic characteristics. Two factors were significantly related with a reduction in support for children’s physical activity. Mother’s self-reported race of Mexican was related with a 2.16 point decrease in social support (*p* = 0.039) from school-year to summer when compared to mothers reporting their race as Mexican American or Hispanic, after adjusting for covariates. Mothers working full-time was associated with a 2.50-point decrease in social support from school-year to summer (*p* = 0.023) as compared to mothers working part-time or unemployed after adjusting for covariates. Slight mother-child agreement was observed for children’s parental social support received and mother’s parental support provided for physical activity during the school-year (median κ = 0.08) and summer (median κ = 0.11). See Table 2 for kappa coefficients comparing mother-child agreement for all items.

## Discussion

The present study expands our current understanding of physical activity in *colonias* populations by examining seasonal differences (school-year to summer) of environmental factors shown to be related with physical activity. As hypothesized, seasonality had an effect on perceived environmental barriers, where they increased from school-year to summer. Given the extreme summer heat in South Texas, it is not surprising that barriers to physical activity increase during the summer. This is consistent with a recent review that found 29 out of 35 studies reported seasonal variation in physical activity among children and adolescents [42]; with the majority of least active seasons or months being the same season or month that had the most extreme weather conditions (e.g., July in Texas; winter in Northern Canada). Other research has found that school environments do not contribute to weight gain as much as non-school environments, as measured by an increase in BMI from school-year to summer [15]. The established propensity for children to gain weight during the summer months could potentially be the result of the increase in perceived barriers to physical activity from school-year to summer seen in this sample.

While previous studies have established that seasonal differences exist in children’s physical activity, this study adds to the current body of literature by identifying associations between seasonality and perceived environmental barriers, opportunities, and support. In this sample, higher total household income and a greater household size were related with greater barriers for children’s physical activity. Previous research has found mixed relationships between economic status and children’s physical activity levels [43, 44]. For children living in *colonias*, greater family income may indicate a working parent or working parents that may not be able to provide transportation or other means of accessing physical activities. A greater household size may indicate less individualized attention for children, and fewer physical activity resources, such as appropriate clothing or shoes for each child. Mother’s significant other’s employment was associated with a decrease in barriers from school-year to summer months. This is a particularly interesting finding given that employment often indicates greater income, which was already shown to increase barriers to physical activity. Instead, it is possible that with a significant other working, the child’s mother may be at home and able to provide transportation to physical activity resources, and be able to accompany the child during physical activities.

While barriers increased during summer months, children’s opportunities for physical activity also increased significantly, contrary to the author’s hypothesis. The concurrent increase in both barriers and opportunities may be related with physical activity in several ways. First, it has been established that children often gain weight in the summer [15]. It is therefore possible that barriers to physical activity (e.g., extreme heat, lack of encouragement, lack of appropriate environment, etc.) outweigh the increase in opportunities for physical activity (e.g., kids playing, informally made soccer fields, etc.) contributing to this phenomenon. It has also been shown that children’s physical activity levels increase in the summer, but so do weight gain and BMI [14]. This may alternatively indicate that barriers may not outweigh opportunities, but that other health behaviors, such as food intake, could be contributing to summer weight gain despite the increase in opportunities for physical activity. Future research should explore this balance between barriers and opportunities, and their combined effect on physical activity participation. Approaching statistical significance, increased opportunities were related with mothers born in the U.S. (vs. Mexico) which could be in part due to the acculturation process. It is possible that mothers born in the U.S. are more familiar with summer needs of children in the U.S. versus mothers born in Mexico. Future research should examine potential cultural differences in summer opportunities in Mexico and the U.S. to identify future intervention needs.

Although not significant, social support for physical activity decreased slightly from the school year to summer months. Parental support is a known positive correlate of physical activity participation in children, but the slight decrease from school year to summer is to our knowledge a novel finding [45]. Although barriers to physical activity increase in the summer, and the extreme summer temperatures likely playing a large role in that increase, other school-specific barriers such as homework become irrelevant. Given this and the increase in opportunities for physical activity over the summer, it should be possible for mothers to offer intangible means of social support to their children (e.g., encouraging children to be more active). The mothers who are home during the day could also provide the other intangible means of social support measured in this study by being active with their children. Mothers that are able to provide transportation to their children would have access to an even greater number of resources for physical activities. Despite the increase in opportunities, slight decreases in social support for physical activity between school-year and summer may be harmful for children living in *colonias* given the potential need for additional support in the summer. Two interesting relationships were identified that should also be further investigated. Children with mothers reporting Mexican race/ethnicity (versus Hispanic or Mexican American – open-ended response) had a significant decrease in social support from school-year to summer, which could be explained by considering cultural differences between these self-identified ethnicities. Future research should explore these potential differences as the present study did not collect adequate information to further examine this. Children with mothers employed full-time at baseline also had decreases in social support from school-year to summer, which could be explained when considering the amount of available time of mothers working full-time as compared to mothers not working or only working part-time. This is potentially accentuated in summer months when children are in greater need of tangible support given their extra discretionary time during the summer. Better understanding of the relationship between these factors and social support may aid in planning more appropriate initiatives to increase social support for physical activity and ultimately increase physical activity levels.

The secondary aim of this study, which was to examine mother-child agreement across the factors, resulted in another important finding. As evidenced by slight and fair median kappa coefficient ratings, discordance between mother’s and child’s perceptions was found in all categories; barriers to physical activity, opportunities for physical activity, and social support for physical activity. Differences between mother’s and child’s perceptions have been found in other fields (e.g., community violence and food security status; [46, 47]), but this study is among the first to examine mother-child discordance regarding perceived physical activity environments. The items with the strongest mother-child agreement included the barrier of having asthma, and having the at-home opportunities of a swing set, trampoline, and a functional basketball hoop. Three of the top four items with the strongest agreement were physical activity opportunities within the home. Across both the school-year and the summer, mother-child agreement was much more likely for home opportunities as compared with neighborhood opportunities. This may be because children and mothers can easily identify whether or not there is a swing set or trampoline in their home or backyard, but may be less aware of other neighborhood resources, especially resources they may not use. Both perceptions of the mothers and children should be considered in efforts to examine and understand physical activity behaviors, given the distinct perceptions between mother-child dyads. Only addressing the concerns of either mothers or children, based on this data, would likely result in an incomplete approach, thus hindering potential effectiveness. Future research that includes environmental perceptions should measure and/or account for mother-child or parent-child differences.

## Conclusions

Despite limitations of this study, these results can be used to further research on the relationships between environmental factors and physical activity; and ultimately help guide efforts to increase physical activity in this population. However, several limitations do need to be considered. For instance, causation could not be examined due to the cross-sectional study design. School year surveys were also completed between February and May of 2011. It is possible that differences in perceived barriers, opportunities, and social support may be present across the months that were not accounted for by using this range. In addition, neither mother nor child physical activity was measured in this study. The effects that seasonal increases in perceived barriers, opportunities, and social support have on physical activity participation could therefore only be speculated. Future research should examine these relationships in a similar model. Lastly, this study was only conducted in *colonias* in Hidalgo County, Texas. Findings are generalizable to other *colonias* populations similar in environments, resources, and demographics, but may not be generalizable to all *colonias* populations across the entire U.S.-Mexico border or other populations. Despite these limitations, this study contributes to the literature by extending our understanding of the perceived barriers, perceived opportunities, and social support for physical activity from the school-year to summer months for children living in *colonias*.

Understanding the effect of seasonality on environmental barriers, opportunities, and social support for physical activity faced by *colonias* residents can be particularly useful to researchers working with *colonias* and with other Hispanic immigrants living in the U.S. Similar to the demographic findings in this study although not as severe, approximately 42% of Hispanic women living in the United States aged 16 and over are unemployed, and 40% of Hispanic men and women are experiencing long-term unemployment [48]. Additionally, when compared to other racial groups, Hispanics in the U.S. report the lowest percentages of those with a high school diploma; only 61% [49]. Most Hispanic and specifically Mexican immigrant families are faced with similar challenges as families residing in *colonias*. These include severe poverty, underemployment, educational disadvantages, increased risk for chronic diseases, and residences within ethnic enclaves. The challenges faced by residents in *colonias* are therefore representative of many challenges faced by Mexican immigrants across the U.S. and can serve as an example for researchers, policy makers, and public health workers when trying to best serve these populations.

## References

[1] Pearson N, Biddle SJH (2011). Sedentary behavior and dietary intake in children, adolescents, and adults: a systematic review. Am J Prev Med.

[2] Biddle SJ, Gorely T, Stensel DJ (2004). Health-enhancing physical activity and sedentary behaviour in children and adolescents. J Sports Sci.

[3] Tremblay MS, Colley RC, Saunders TJ, Healy GN, Owen N (2010). Physiological and health implications of a sedentary lifestyle. Appl Physiol Nutr Metab.

[4] Must A, Strauss RS (1999). Risks and consequences of childhood and adolescent obesity. Int J Obes Relat Metab Disord.

[5] Tremblay MS, LeBlanc AG, Kho ME, Saunders TJ, Larouche R, Colley RC, Goldfield G, Gorber S (2011). Systematic review of sedentary behaviour and health indicators in school-aged children and youth. Int J Behav Nutr Phys Act.

[6] U.S. Department of Health and Human Services (USDHHS) (2010). Healthy People 2020. Washington, DC: U.S. Government Printing Office.

[7] Van Der Horst K, Paw MJCA, Twisk JWR, Van Mechelen W (2007). A brief review on correlates of physical activity and sedentariness in youth. Med Sci Sports Exerc.

[8] Center for Disease Control and Prevention (CDC): **Youth Risk Behavior Surveillance- United States, 2013.** *MMWR Morb Mortal Wkly Rep* Retrieved from http://www.cdc.gov/HealthyYouth/yrbs/index.htm

[9] Davison KK, Lawson CT (2006). Do attributes in the physical environment influence children’s physical activity? a review of the literature. Int J Behav Nutr Phys Act.

[10] Tucker P, Gilliland J (2007). The effect of season and weather on physical activity: a systematic review. Public Health.

[11] Merrill RMS, Eric C, White GL, Druce D, Shields EC (2005). Climate conditions and physical activity in the United States. Am J Health Behav.

[12] Plasqui G, Westerterp KR (2004). Seasonal variation in total energy expenditure and physical activity in Dutch Young Adults. Obes Res.

[13] Merchant AT, Dehghan M, Akhtar-Danesh N (2007). Seasonal variation in leisure-time physical activity among Canadians. Can J Public Health Rev Can Santé Publique.

[14] Baranowski T, O’Connor T, Johnston C, Hughes S, Moreno J, Chen T-A, Meltzer L, Baranowski J (2014). School year versus summer differences in child weight gain: a narrative review. Child Obes Print.

[15] Von Hippel PT, Powell B, Downey DB, Rowland NJ (2007). The effect of school on overweight in Childhood: gain in body mass index during the school year and during summer vacation. Am J Public Health.

[16] **The Weather Channel, LLC** 2014. http://www.wunderground.com

[17] Umstattd Meyer MR, Sharkey JR, Patterson MS, Dean WR (2013). Understanding contextual barriers, supports, and opportunities for physical activity among Mexican-origin children in Texas border colonias: a descriptive study. BMC Public Health.

[18] Donelson AJ, Esparza AX (2010). The Colonias Reader: Economy, housing, and public health in U.S.-Mexico border colonias.

[19] Ward PM (1999). Colonias and Public Policy in Texas and Mexico: Urbanization by Stealth.

[20] Vijayaraghavan M, He G, Stoddard P, Schillinger D (2010). Blood pressure control, hypertension, awareness, and treatment in adults with diabetes in the United States-Mexico border region. Rev Panam Salud Publica.

[21] United States-Mexico Border Area (2007). Health in the Americas.

[22] Humes KR, Jones NA, Ramirez RR, Humes KR, Jones NA, Ramirez RR (2011). Overview of race and Hispanic origin: 2010. U. S. Census Bureau.

[23] Jensen L (2006). New immigrant settlements in rural America: Problems, prospects, and policies. Carsey Institute, University of New Hampshire.

[24] Centers for Disease Control and Prevention (CDC) Division of Diabetes Translation (2009). Percentage of risk factors for complications among adults with diabetes, United States, 2007. Department of Health and Human Services, Centers for Disease Control and Prevention.

[25] Parks SE, Housemann RA, Brownson RC (2003). Differential correlates of physical activity in urban and rural adults of various socioeconomic backgrounds in the United States. J Epidemiol Community Health.

[26] U.S. Census Bureau: **State and County QuickFacts.** Data derived from Population Estimates, American Community Survey, Census of Population and Housing, State and County Housing Unit Estimates, County Business Patterns, Nonemployer Statistics, Economic Census, Survey of Business Owners, Building Permits. Last Revised: Tuesday, 08-Jul-2014 06:46:03 EDT. http://quickfacts.census.gov/qfd/states/48/48215.html (accessed July 15, 2014)

[27] Dean WR, Sharkey JR, Johnson CM, St John J (2012). Cultural repertoires and food-related household technology within colonia households under conditions of material hardship. Int J Equity Health.

[28] Sharkey JR, Dean WR, John JAS, Huber JC (2010). Using direct observations on multiple occasions to measure household food availability among low-income Mexicano residents in Texas colonias. BMC Public Health.

[29] Sharkey JR, Dean WR, Johnson CM (2011). Association of household and community characteristics with Adult and child food insecurity among Mexican-Origin households in Colonias along the Texas-Mexico Border. Int J Equity Health.

[30] Sharkey JR, Nalty C, Johnson CM, Dean WR (2012). Children’s very low food security is associated with increased dietary intakes in energy, fat, and added sugar among Mexican-origin children (6-11 y) in Texas border Colonias. BMC Pediatr.

[31] Sharkey JR, Horel S, Han D, Huber JC (2009). Association between neighborhood need and spatial access to food stores and fast food restaurants in neighborhoods of Colonias. Int J Health Geogr.

[32] Brownson RC, Baker EA, Housemann RA, Brennan LK, Bacak SJ (2001). Environmental and policy determinants of physical activity in the United States. Am J Public Health.

[33] Gordon-Larsen P, Griffiths P, Bentley ME, Ward DS, Kelsey K, Shields K, Ammerman A (2004). Barriers to physical activity: qualitative data on caregiver–daughter perceptions and practices. Am J Prev Med.

[34] Salmon J, Owen N, Crawford D, Bauman A, Sallis JF (2003). Physical activity and sedentary behavior: a population-based study of barriers, enjoyment, and preference. Health Psychol.

[35] Umstattd MR, Baller SL, Hennessy E, Hartley D, Economos CD, Hyatt RR, Yousefian A, Hallam JS (2012). Development of the Rural Active Living Perceived Environmental Support Scale (RALPESS). J Phys Act Health.

[36] Sallis JF, Glanz K (2009). Physical activity and food environments: solutions to the obesity epidemic. Milbank Q.

[37] Yousefian A, Hennessy E, Umstattd MR, Economos CD, Hallam JS, Hyatt RR, Hartley D (2010). Development of the rural active living assessment tools: measuring rural environments. Prev Med.

[38] Ball K, Jeffery RW, Abbott G, McNaughton SA, Crawford D (2010). Is healthy behavior contagious: associations of social norms with physical activity and health eating. Int J Behav Nutr Phys Act.

[39] Grieser M, Neumark-Sztainer D, Saksvig BI, Lee J, Felton GM, Kubik MY (2008). Black, Hispanic, and white girls’ perceptions of environmental and social support and enjoyment of physical activity. J School Health.

[40] Trost SG, Sallis JF, Pate RR, Freedson PS, Taylor WC, Dowda M (2003). Evaluating a model of parental influence on youth physical activity. Am J Prev Med.

[41] SAS (Version 9.3) (2012). SAS Institute Inc.

[42] Carson V, Spence JC (2010). Seasonal variation in physical activity among children and adolescents: a review. Pediatr Exerc Sci.

[43] Raudsepp L (2006). The relationship between socio-economic status, parental support and adolescent physical activity. Acta Paediatr.

[44] Duncan M, Woodfield L, Al‒Nakeeb Y, Nevill A (2002). The impact of socio‒economic status on the physical activity levels of British Secondary School Children. Eur J Phys Educ.

[45] Sallis JF, Prochaska JJ, Taylor WC (2000). A review of correlates of physical activity of children and adolescents. Med Sci Sports Exerc.

[46] Hill HM, Jones LP (1997). Children’s and parents’ perceptions of children’s exposure to violence in urban neighborhoods. J Natl Med Assoc.

[47] Nalty CC, Sharkey JR, Dean WR (2013). Children’s reporting of food insecurity in predominately food insecure households in Texas border colonias. Nutr J.

[48] United States Department of Labor (2011). The latino labor force in the recovery.

[49] Ryan CL, Siebens J (2012). Educational Attainment in the United States: 2009. United States Census Bureau, U.S Department of Commerce, Economics and Statistics Administration.

[CR50] The pre-publication history for this paper can be accessed here: http://www.biomedcentral.com/1471-2458/14/958/prepub

